# High mortality in Brazilian intensive care units can be a problem of laws rather than a technical one: focus on sedation practices

**DOI:** 10.5935/2965-2774.20230337-en

**Published:** 2023

**Authors:** Cassiano Teixeira

**Affiliations:** 1 Department of Internal Medicine and Rehabilitation, Universidade Federal de Ciências da Saúde de Porto Alegre - Porto Alegre (RS), Brazil

## TO THE EDITOR

In the care of ventilated critically ill patients, there is a consistent relationship between deeper sedation and worse intensive care unit (ICU) outcomes.^(1,2)^ Deep sedation in the first 48 hours of an ICU stay has been associated with delayed time to extubation, higher need for tracheostomy, longer ICU stays, and increased risk of hospital and long-term death.^(3)^ This association in patients with acute respiratory distress syndrome (ARDS) and other severely ill patients is of particular concern. In this sense, perhaps the greatest advances in critical patient care can be summarized by the ABCDEF bundle in critical care (Assess, prevent, and manage pain; Both spontaneous awakening trials and spontaneous breathing trials; Choice of analgesia and sedation; Delirium-assess, prevent, and manage; Early mobility and exercise; and Family engagement and empowerment); in this approach, light sedation as opposed to deep sedation seems to be preferred.^(4,5)^ Each individual component of the bundle is evidence-based and has been validated in multiple clinical trials. The bundle combines the individual impact of each intervention into a synergistic process of care that improves ICU outcomes and can mitigate the burden of postintensive care unit syndrome in survivors. Authors have already demonstrated improving both short-term need (length of delirium, need for physical restraints, days on mechanical ventilation) and long-term outcomes (ICU readmission, discharge to facility) in critically ill patients when these recommendations are practiced.^(4)^

In recent years, high-income countries have shown an important reduction in the mortality of critically ill patients, a fact that has not been replicated in lowand middle-income countries. However, why does it not occur? Let us evaluate critical illness data in Brazil, a continental and multicultural country that has failed systematically to reduce critical illness, morbidity and mortality. Recent well-conducted randomized clinical trials (RCTs) in Brazil^(6,7)^ have demonstrated the inability of some clinical teams to achieve the internationally recommended goals of light sedation. The CHECKLIST trial^(6)^ (n = 6,877), including any patients admitted to adult ICUs, showed low adherence (control group, 35.0% *versus* intervention group, 40.5%, p = 0.05) of the ICU staff in providing moderate sedation to alert and calm patients (Richmond Agitation-Sedation Scale - RASS -3 to 0). Patient´s in-hospital mortality (truncated at 60 days) was 33.9% (mean Simplified Acute Physiology Score 3 - SAPS 3 at admission, 51.2 [standard deviation - SD, 17.9] in the control group and 54.2 [SD, 17.5] in the intervention group). Another RCT, the Acute Respiratory Distress Syndrome Trial (ART)^(7)^ (n = 1,010), which evaluated patients with moderate to severe ARDS, showed that 96.8% of the control group and 73.3% of the intervention group (p < 0.001) needed neuromuscular blockage (a proxy of deep sedation practice), with a global patient mortality of 52.3%. This practice of deep sedation seems to be more common in lowand middle-income countries.^(8)^ However, in high-income countries, a multinational PRactice of VENTilation patients (PROVENT) trial (studying 2,377 ARDS patients in ICUs in 50 European countries) showed that only 21.7% of patients received neuromuscular blockage; mortality rates of 40.3 to 46.1% were found for moderate and severe ARDS, respectively.^(9)^ Ratifying the numbers, a multicenter Brazilian Epidemiology of Respiratory Insufficiency in Critical Care (ERICC) study evaluated 773 adult patients admitted to 45 ICUs and showed a hospital mortality of 52% among ARDS patients;^(10)^ recently, the analysis of the first 254,288 COVID-19 patients admitted to Brazilian hospitals showed a mortality of 80% among those who needed invasive mechanical ventilation.^(11)^

Brazilian intensive care is recognized worldwide for its competence and quality. However, why has a significant reduction in ICU mortality not occurred over the years? The explanation may be centered more on legal than technical labor aspects. The current comprehension and interpretation of some labor laws may impose barriers to carrying out truly organized and multidisciplinary teamwork targeting patient-centered care, and Brazilian ICU sedation practices could contribute to the high ICU mortality of critically ill patients. Around the world, nurses are encouraged to use nurse-driven sedation scales, such as the Sedation Assessment Scale or the Richmond Agitation Sedation Scale, as assessment tools for sedation titration and practice nurse-driven sedation protocols. However, the Brazilian laws recommended some worrying practices, such as “The diagnosis and prescription of medications are acts of exclusive competence of the physician, dentist, and veterinarian, in cases restricted to the respective specialties”. In the field of medicine, the act of the medical professional is regulated by the Federal Council of Medicine (CFM - *Conselho Federal de Medicina*) Resolution 1627/2001.^(12)^ The content of this recommendation, associated with the agreement of the Federal Nursing Council (COFEN), makes it very difficult to practice internationally recommended nurse-driven sedation protocols. The ORCHESTRA study, which enrolled 129,680 patients admitted to 93 Brazilian ICUs, showed that nurses’ autonomy positively influenced patients’ outcomes.^(13)^ However, as expected in Brazil, only 17.2% of the sample was classified in Cluster 3 (with a high grade of nurse autonomy). In addition, throughout the world, critical care nurses are often reluctant to participate in daily awakening trials.^(8,14)^ They fear that patients may become frightened upon awakening and self-extubate, remove invasive lines, or become combative.^(15)^ Regarding these decisions, they do not receive support from nurse managers (based on legal aspects) or from intensive care physicians (based on patient responsibility aspects). Therefore, turning off sedation daily and restarting sedation only if needed at the lowest dose to maintain the chosen target level of consciousness is exclusively a medical decision. Use of bolus sedation dosing requires a medical prescription. Another medical decision may include “Do not wake the patient up every 2 hours to check vital signs, check blood sugar, or repositioning during the night.” The definition of starting a breathing trial needs medical approval. The application of a protocol that incorporates early progressive mobility and exercise depends on medical liberation.

In Brazilian ICUs, it is very common for doctors to receive questions such as “Doctor, can I apply an antipyretic to the patient?” “Doctor, the patient is agitated, can I apply bolus of midazolam?” “Doctor, what patients can I get out of bed today?” “Doctor, will we have a multidisciplinary round today?” In summary, critical care medicine is centered on the physician, and teamwork is not generally thought of. This behavior of professionals that disrespects the roles of multidisciplinary help denies the importance of nurses, physiotherapists, nutritionists, dentists, speech therapists, and psychologists. Further, the training of health professionals does not encourage interdisciplinarity. These are isolated academic formations, sometimes even with hostile refinements. The classes of the courses are separated, with no exchange of experiences between professionals and with rare cases of one specialty administering a class to another.

Thus, a major cultural and responsibility shift could potentially significantly benefit the country’s critically ill patients. Increasing autonomy in decision-making by nursing and physiotherapy in ICUs could bring great benefits in terms of facilitating multidisciplinary work and possibly improving clinical outcomes for critically ill patients in Brazil. The sedation protocol for critically ill patients urgently needs to be in different hands.
